# Predictive modelling and experimental analysis of radiation-resistant MXene–silicon heterojunction solar cells

**DOI:** 10.1186/s11671-026-04932-9

**Published:** 2026-09-14

**Authors:** Deepthi Jayan K, Elsaeedy H I, Krishna Prasad S, Gabr Goshu Syum

**Affiliations:** 1 Department of Basic Sciences and Humanities, Rajagiri School of engineering & Technology (Autonomous), Kochi, 692 039 Kerala India; 2 Department of Physics, College of Science, King Khalid University, Abha, 61413 Saudi Arabia; 3 Nitte (Deemed to be university), NMAM Institute of Technology (NMAMIT), Department of Mechanical Engineering, Nitte, Mangalore, India; 4 Faculty of Mechanical and Industrial Engineering, EiT-M, Mekelle University, P. O. Box 231, Mekelle, Tigray, 7000 Ethiopia

**Keywords:** MXene (Ti_3_C_2_T_X_)–Si, Solar cells, Radiation tolerance, Power conversion efficiency, Digital twin

## Abstract

MXene-silicon heterojunction solar cells (Ti_3_C_2_T_x_-Si) have become a potential candidate for radiation-tolerant photovoltaic (PV) applications, specifically in harsh space conditions. In this study, a combination of predictive modeling and experimental verification is used to evaluate the degradation characteristics of Ti_3_C_2_T_x_ - Si solar cells under proton and electron irradiation. The study indicates that Ti_3_C_2_T_x_ and Si heterojunction devices can be used to achieve a superior yield, maintaining over 84% of the original power conversion efficiency (PCE), as compared to conventional Si solar cells that can only maintain 55% efficiency under the same exposure. X-ray diffraction (XRD), Raman spectroscopy, and Atom Probe Tomography (APT) reveal the interfacial stability of the MXene layers that alleviates the radiation induced structural damage. A physics-based digital twin (DT) framework is applied to the synthetic degradation data, which achieved a high prediction accuracy of 90% (R^2^ > 0.96) using a training model based on Random Forest Regression (RFR), which in turn can aid to forecast the device performance with better accuracy. The PV performance parameters like the open circuit voltage (V_OC_), short circuit current density (J_SC_), fill factor (FF) and PCE are studied as functions of mission-relevant exposure durations. This hybrid approach of machine learning (ML)-aided modeling, combined with in situ experimental verification develops a solid framework for real-time health monitoring and lifetime prediction of PV devices, making it highly suitable for deployment in radiation-intensive orbital environments.

Clinical Trial Registration: Not applicable. This study is not a clinical trial and does not involve any medical intervention or human subjects.

## Introduction

Considering that the interest in long duration and high efficiency harsh missions is still increasing, solar energy is the most dependable and long-term facilitating power source, which has the ability to offer steady and a virtually unlimited source of energy in harsh environment without any limits even at high powers [1]. Power infrastructure and power systems have typically relied on crystalline silicon (C-Si) photovoltaic (PV) systems, which have now a well-established fabrication technology, making the systems relatively inexpensive and having well-understood performance measures [2]. This is, however, hindered by harsh conditions e.g. interminable exposure to high-energy solar proton events, galactic cosmic rays, and radiation, which are extremely hostile to Si-based solar cells [3, 4]. Such energetic particles expel atoms in an atomic grid to produce deep-level traps and point defects that eventually decrease the photon assimilation, carrier life, and overall stagger of transformation [5, 6]. Energies of proton (10–100 MeV) and electron (0.1-1 MeV) have been of special adverse effects to Si because they have a high tendency of causing bulk damage [7–9]. Radiation hardening techniques even at a traditional Si PV asset such as radiation-hardened c-Si cells, protective cover glasses, and power redundancy architectures are unable to eliminate performance degradation during sustained radiation flux [10, 11].

Typical ground-based radiation testing techniques will not usually reproduce the full range of in-orbit degradations because they do not include long term and cumulative damage effects in time-dependent irradiation environments [12]. Hence, there is a pressing concern to establish high performing materials and composites, which have high radiation tolerance and endurance of functionality over longer missions [13]. In this regard, two-dimensional (2D) materials like MXenes, especially Ti_3_C_2_T_X_, are considered as a very promising candidate in the next-generation PV cells. The surface termination groups (X = O, OH, F) and functional terminal groups (T_X_ = - O, - OH, - F) enable the MXenes to have metallic conductivity, hydrophilic surfaces, and the flexibility of mechanical properties, in addition to their great electrochemical stability and tunable optoelectronic properties [14, 15]. The heterostructures enabled by MXene combinations with Si yield a versatile set of high quality Schottky or pseudo-ohmic oppositions that encourage fast carrier segregation, reduced recombination at the surface, and better band matching at and close to the interface [16]. Furthermore, the possibility of radiation shielding possessed by MXenes due to high atomic density and a layered structure offers a distinctive benefit for MXene over such traditional nanomaterials [17]. The first experiment has also shown that MXene-Si junctions can retain notable PV performance against simulated irradiation and are thus, also suited to radiation-tolerant power generation in underlying harsh environment [16, 18].

Recently, Liu et al. optimized c-Si–MXene back-heterojunction devices for comprehensive performance [19], while Li et al. introduced silver-nanowire (Ag NW)-assisted MXene electrodes to improve the conductivity and scalability of Si heterojunction cells [20]. In parallel, Agresti et al. showed that titanium-carbide MXenes enable precise work-function and interface engineering in PV devices [21]. Regarding the harsh-environment operation, Vogl et al. demonstrated that devices based on 2D materials retain functionality under space-relevant radiation [22], and complementary studies on perovskite [10], organic [23], and combined electron/proton-irradiated cells [24] have mapped the radiation response of competing PV technologies. However, to the best of our knowledge, no prior study has combined the irradiation of MXene–Si heterojunction cells with atomistic simulation and machine learning (ML)-based lifetime prediction, which is the gap addressed in the present work.

MXene has a great zest to withstand in the gross environments like high-energy proton and electron radiations, and is very appropriate to the harsh application, especially the Ti_3_C_2_T_x_ MXene. Recent experimental studies proved that MXenes still possessed consistent layers, electrical conductivity, and surface terminations after a long period of exposure to the radiation [25, 26]. Ti_3_C_2_T_x_ - Si heterojunctions have emerged to have good PV potential with a PCE of over 17%, and possess good thermal and mechanical stability [18, 26]. They can be designed with 2D form; tunable work function, and high carrier mobility offering efficiency in charge separation and transport across the junction, maximizing their operation and minimizing degradation. PV systems based on MXenes, therefore, have the revolutionary capability to run satellites, orbital platforms and rovers on other planets, as summarized in Table 1.

**Table 1:** Useful properties of MXene for the enhancement of efficiency of solar cells [25–28]

Property	Typical value / Characteristics	Efficiency benefit
Electrical conductivity	~ 10^6^ S/m	Low resistive losses, improved charge collection [25]
Optical transparency	~ 97.7% (monolayer)	Allows lighter to reach silicon absorber [25]
Thermal conductivity	~ 55.8 W/m·K	Prevents thermal damage under irradiation [26]
Carrier mobility	> 10^4^ cm^2^/V·s	Fast carrier transport, reduced recombination [27]
Mechanical strength	~ 0.3TPa TPa (Young’s modulus)	Maintains interface integrity under stress [28]
Chemical stability	High resistance to oxidation	Preserves material quality in harsh environments [22]
Thickness	1 nm (monolayer)	Minimizes shading, enables tunneling contact [29]
Work function tunability	4.5–4.8 eV (tunable via doping)	Enhances junction performance with silicon [21]

Reliable PV power is the backbone of space missions, yet harsh environment displace atoms in the Si lattice, creating deep-level traps that progressively degrade carrier lifetime, junction quality, and PCE [3–8]. Ground-based accelerated testing cannot reproduce the cumulative, time-dependent, multi-stressor degradation experienced over years in orbit, so end-of-life performance must be extrapolated rather than measured [12]. 2D Ti_3_C_2_T_x_ MXenes, combining metallic conductivity and chemical robustness, offer a hardware-level solution by shielding the junction region against recoil cascades and gettering radiation-generated defects, while retaining the low-cost Si platform [14, 25]. However, existing MXene–Si studies address only beginning-of-life efficiency and no prior work has combined mission-relevant irradiation with a predictive lifetime model.

The main aim of the study is to settle the question of radiation tolerance and stability of the predictive performance of MXene-Si heterojunction solar cells in simulated near-harsh conditions. Bearing the drawbacks of the purely experimental testing related to the inability to simulate the long-term orbital exposure, a fully-fledged digital twin (DT) environment is designed to model the real-time operating behaviour of the device under irradiation. DTs have found application harsh systems as instrumentation in the delivery process of predictive diagnostics and performance optimization as well as mission assurance [29, 30]. DTs used to monitor PV systems will enable real-time monitoring of how materials age and develop defects and how radiation causes performance drifts in the systems through inclusion of physics-based models and synthetic data [31–33].

This study presents MXene-Si heterojunction solar cells, which are subjected to empirical exposure tests as well as a digital simulation of the same conducted through radiation transport and degradation modelling. The third framework (i.e. DT) takes advantage of sophisticated ML models, e.g. Random Forest Regression (RFR), learning on synthetic data, to predict degradation metrics, open circuit voltage (V_OC_), short circuit current density (J_SC_), fill factor (FF), and PCE. Such reports are tested against experimental datasets with fidelity R^2^ exceeding 0.96. Combination of next-generation nanomaterials and DT-enabled predictive analytics will provide the basis of future PV systems designed with long lifespan, autonomous resilience, and mission-assured power reliability.

## Materials and methods

### Fabrication of MXene - Si heterojunction solar cells

The heterojunction solar cells were prepared with monocrystalline n-type Si wafers with a thickness of 300 μm and resistivity of 1–10 Ω.cm as the PV absorber layer. Si wafers used before integration were cleaned in uniform RCA cleaning process and washed in diluted hydrofluoric acid (HF) that eliminated the native surface oxides and passivated the surface [34]. After selective etching of Al layers of the Ti_3_AlC_2_ MAX phase using a LiF-HCl solution, according to the known protocols of obtaining the minimal defective multilayer MXenes, the Ti_3_C_2_T_X_ MXene was obtained [35]. The Ti_3_C_2_T_X_ produced after etching was delaminated through sonication and centrifugation with deionized (DI) water to produce few-layer dispersion that could then be used to produce the devices [36]. This was vacuum-filtrated and transferred onto the previously cleaned Si surface via a dry-press, or spin-aided transfer method, which guarantees conformal contact, and contamination at the interface between the two surfaces. To achieve a better electrical contact and interconnect, samples were annealed in an inert (Ar) or forming gas (5% H_2_ in Ar) atmosphere at approximately 300 °C for 30 min [37]. A 50 nm thick, indium tin oxide (ITO), was deposited by RF magnetron sputtering and patterned using a shadow mask. A full-area aluminium film was deposited by electron beam evaporation on the rear contacts and it was thermally annealed at 400 °C to activate ohmic contact [38].

The resulting vertical heterojunction was composed of Ti_3_C_2_T_X_ MXene as transparent conductive electrode and modifier at the interface, whereas n-type-Si played the role of the photoabsorbent layer and source of primary charge carriers [33]. This structure was well suited to enhance carrier collection, suppress interfacial recombination and improve radiation endurance. Twelve 1 cm^2^ devices were produced in ISO Class 5 clean room environment to prevent particulate contamination and oxidation. Measurements were performed to bag devices in nitrogen-blanketed enclosures before electrical and radiation evaluation tests to maintain the integrity of materials and avert atmospheric destruction.

**Fig. 1:**
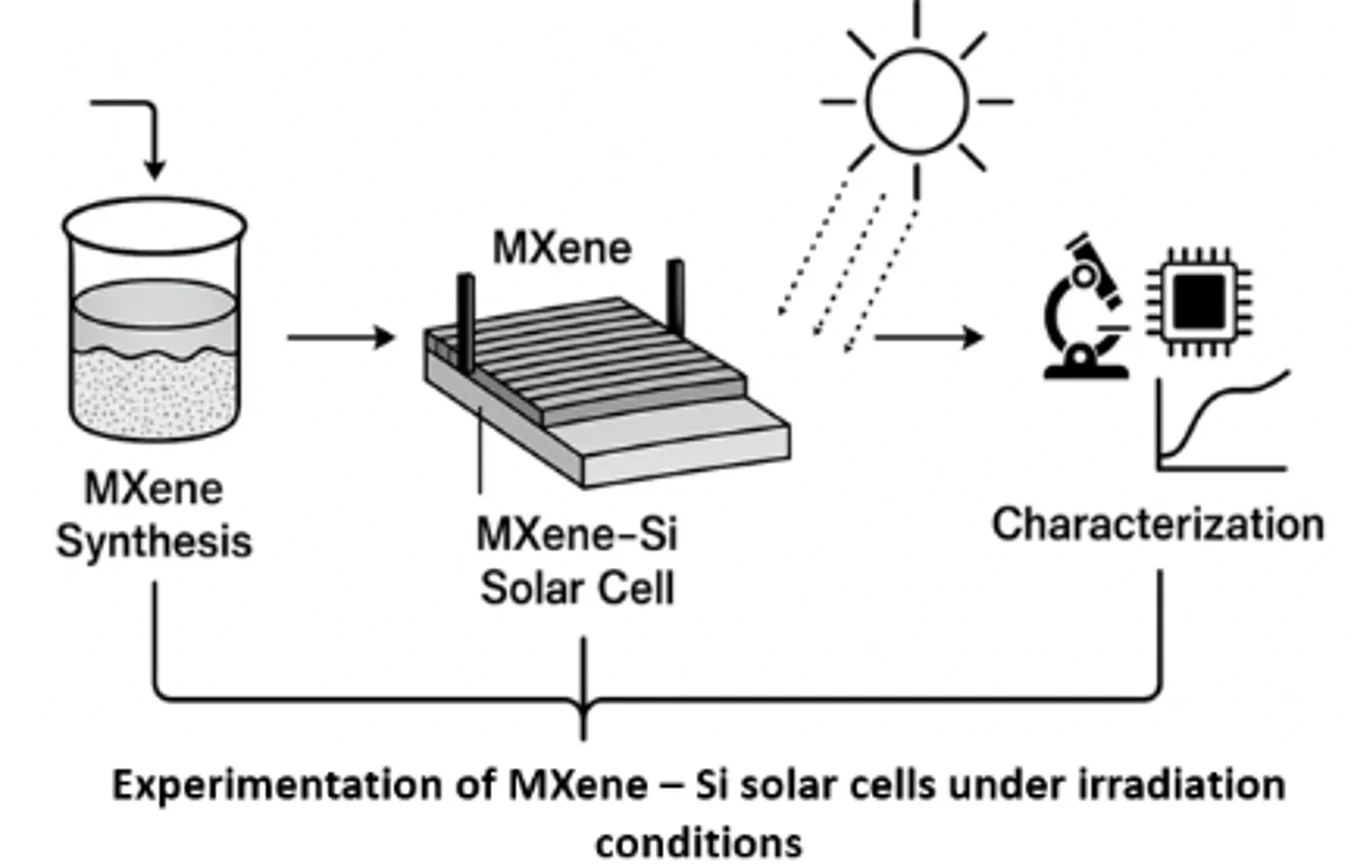
Schematic representation of the experimental workflow for evaluating MXene– Si solar cells under irradiation conditions

Figure 1 describes the workflow of synthesis, irradiation and characterization of MXene-Si heterojunction solar cell. At first, Ti_3_C_2_T_x_ MXene nanosheets (NSs) are fabricated through preferential etching and delamination reactions. The MXene-Si heterojunction device is thereafter made by transferring these flakes onto pre-cleaned monocrystalline Si substrates. The artificial cells are then subjected to simulated harsh radiation i.e. to the high energy protons and electrons, via a controlled radiation chamber that resembles the environment of harsh and low earth orbit (LEO). After irradiation, the devices are tested with a calibrated solar simulator in standard AM 1.5G conditions. A source-measure unit and data acquisition systems are used to obtain PV parameters, such as V_OC_, J_SC_, FF and PCE. The overall experimental procedure allows a thorough evaluation of the radiation effects on the degradation of the device that provides predictive understanding of the life and reliability of the devices in the extra-terrestrial environment.

To investigate the changes in the structural and material integrity of the MXene-Si heterojunction solar cells before and after chained irradiation, a blend of X-ray diffraction (XRD) and Raman spectroscopy were utilized. The results of the XRD studies supported the accuracy of the structure and phase retention of the composite since it presented clear crystallographic planes of reflection belonging to silicon (Si (111), Si (220), and Si (311)) and MXene (002). Raman spectroscopy was also used to confirm the crystallinity and states of the bonding whereby the G and 2D bands gave details on the ordering of lattices as well as defects that existed in the MXene layer. High-quality layered structures were confirmed by pre-irradiation spectra, whereas post-irradiation shifts and peak broadening pointed out to a possible formation of defects and disorder.

Along with the experimental results, a physics-informed DT model was trained to mimic the change of interface when exposed to radiation. Large-scale atomic/molecular massively parallel simulator (LAMMPS) was used to model the atomistic-level interactions between the layers of MXene and Si. The slab of c-Si (100) was used as the base substrate, and the sheet of Ti_3_C_2_T_x_ MXene is mounted on the top of it, forming the heterojunction interface. The visualization of simulation results in terms of atomic displacement, defect evolution, and interfacial mixing was carried out with the help of Open Visualization Tool (OVITO) that allowed the acquisition of quantitative information of damage development at the atomic scale. This DT methodology was used as a predictive analysis that matched the experiment-based pathways of degradation with structural changes [39].

A novel and robust DT framework was established to explore the effect of radiation-induced degradation and the evolution of performance of MXene-Si heterojunction solar cells under simulated harsh radiation conditions. LAMMPS atomistic codes with a hybrid force field code were utilized to perform atomistic simulations, where the adaptive intermolecular reactive empirical bond order (AIREBO) potential modelled the carbon-based structure of the MXene surface, with AIREBO being used to describe the carbon part of the MXene structure, and the Embedded Atom Method (EAM) being used to describe the interaction in between the Si lattice. In the z-axis direction, simulation was set with fixed boundary conditions and along the x and y directions, periodic boundary conditions were imposed. The atomic overlaps have been solved through initial energy minimization after which NVT equilibration was conducted at room temperature of 300 K with time step of 1 fs and a simulation length of about 10,000–50,000 steps depending on the phenomenon under investigation [19].

Thermal cycling was imposed by incrementally heating the system between 300 K and 800 K, but radiation damage was modelled by allowing the virtual atomic displacements and ion impact processes to generate point defects and interfacial mixing. OVITO was employed to perform the post-processing of simulation outputs, which utilizes coordination analysis, cluster tracking, and visualization of atomic diffusion by a color-coded mapping of C, Si and O species to monitor the structural evolution. The physical assumptions within the DT were validated via comparing the extracted results in the form of 1D compositions and defect density trends with the real-world parameters.

The predictive model was subsequently expanded by a RFR algorithm that was trained on an artificial degradation dataset created based on the simulation results. The dominant inputs were radiation fluence, exposure time and thermal stress (40 °C). This model accurately predicted the change of main parameters of PV cells such as V_OC_, J_SC_, FF, and PCE. Surface plots of parameters were constructed and formulae were established to characterize performance degradation on mission related stressors. The digital-experimental hybrid model is a solid means of predicting the in-orbit lifetime of reliability and performance of MXene-based solar cells under intense radiation environments like LEO or harsh environment [40, 41].

The DT is organized in three coupled layers: the atomistic simulation layer (LAMMPS/OVITO), the physics-informed degradation model of Eqs. (1)–(4), and the RFR surrogate. A synthetic degradation dataset of *N* = 5,000 samples was generated by sampling the stressor space - radiation dose 10^4^–10^7^ Gy (logarithmic grid), exposure time 0–200 h, and operating temperature 31–49 °C - through the calibrated degradation equations, with additive Gaussian noise consistent with the ± 4% model uncertainty (ε in Eqs. 1–4). All symbols are defined in Eqs. (1)–(4) and the nomenclature; the vertical axis of panel (a) denotes the modelled V_OC_ (D, t, T) to emulate measurement scatter. Inputs were {log_10_ D, t, T} and outputs {V_OC_, J_SC_, FF, PCE}. 80%/20% train/test split stratified across dose decades; hyperparameters (200 trees, max. depth 12, min. samples per leaf 2) tuned by grid search under 5-fold cross-validation model quality was quantified by the coefficient of determination (R^2^), root-mean-square error (RMSE), and mean absolute error (MAE) on the held-out test set, followed by external validation against the experimentally measured post-irradiation parameters of the fabricated devices; the RFR surrogate achieved R^2^ > 0.96, for all the four outputs.

### Radiation exposure setup

In an effort to evaluate the radiation tolerance of MXene - Si heterostructure solar cells, high energy radiation experiments were systematically done under the simulated harsh settings. The near-vacuum condition of a LEO and geostationary orbit (GEO) environment was simulated using a stainless-steel vacuum chamber with a base pressure of ~ 10^− 5^ Torr. Two types of radiations have been chosen, according to the most widespread in harsh missions: 50 MeV protons as types of solar proton events (SPEs) and 1 MeV electron as the type of trapped electron fluxes in Van Allen belt [42, 43]. Solar cell specimens of a precise active area of 1 cm^2^ were exposed to various degrees of fluence, which included 10^12^ to 10^15^ particles/cm^2^ of the electrons and 10^12^ to 10^14^ particles/cm^2^ in the case of protons. A raster scanning system was used to maintain uniformity of beams over the surface of the device with full time monitoring using Faraday cups and beam profile diagnostics. Calibrated solid-state dosimeters that are located closely to the devices were used to ensure fluence accuracy with an uncertainty margin of +/- 5% [19, 23, 24].

To prevent thermal effects and the radiation response, the samples were mounted on thermally isolated stages to use passive cooling. The temperature was maintained at 20–25 °C during irradiation. The interfacial and material integrity of the devices were ensured by immediately placing them in glove boxes filled with nitrogen after irradiation. The tests were carried out under a close control on safety and operating conditions. Lead shielding encapsulated the radiation chamber and all procedures were carried out remotely so as not to endanger the personnel. Experimental procedures were found to comply with cleanroom protocols of ISO 14,644 and the radiation safety measures of IAEA [18, 44, 45]. This stringent procedure made it possible to measure the impact of radiation induced degradation processes in MXene-Si solar cells in controlled experiments, an important step in determining their exploitation towards harsh PV environment to prevent oxidation or additional ambient degradation before characterization.

**Fig. 2:**
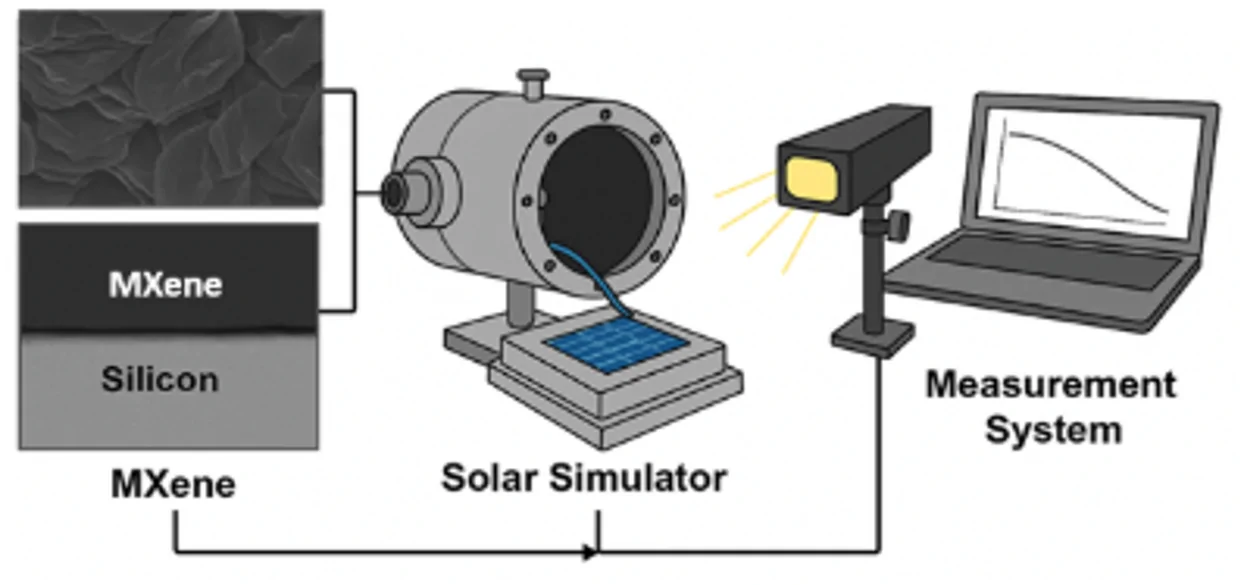
Schematic illustration of experimental setup for PV performance testing of a MXene-Si solar cell under controlled illumination

Figure 2 represents the performance testing of MXene -Si solar cells in simulated harsh environment. It shows the test bench, which is integrated to test the hypothesis that MXene-Si heterojunction solar cells have a higher current density compared to conventional heterojunction solar cells. The experiment is centred on a calibrated solar simulator capable of producing the AM 1.5G solar spectrum, to irradiate the test device under standard and stress induced conditions. The solar cell is mounted on a metallic test fixture securely for ensuring mechanical stability and reliable thermal management during measurement. Electrical connections to the front and rear electrodes are introduced with the help of probes or crocodile clips serving as contact terminals. The current–voltage (I-V) characteristics are then recorded in real time to estimate the PV performance of the device under the applied conditions.

To simulate the possible harsh environmental stress conditions, the platform has an adjacent built-in radiation or vacuum chamber (left), in which the device can be subjected to electrons or protons irradiation with particle energy being 100 keV or thermal cycling at high vacuum (~ 10^− 5^ Torr). The structural integrity of MXene and the evolution of defects can also be analyzed using the non-destructive technique of post-irradiation Raman spectroscopy (bottom right) that monitors variations in characteristic Raman bands, including shifts in peak position, changes in intensity, and band broadening associated with structural disorder and defect formation [18]. An electronic interface connected to a source-measure unit (SMU) tracks the most essential PV characteristics on a continuous basis including V_OC_, J_SC_, FF and PCE. The system can be used to synchronously acquire experimental PV performance data along with the DT based model outputs, thus, enabling comparative degradation studies between simulated lifetime predictions and the real-time response of the devices under artificial stress conditions [46]. This integrated architecture is useful for predictive modelling, performance estimation and design and qualification of the radiation tolerant PV technologies.

### Characterization techniques

To access the effects of the radiation exposure in MXene-Si heterojunction solar cells, rigorous electrical, optical, and structural characterization were done. PV characteristics were examined under calibrated AM 1.5G solar (1000 W/m^2^) simulator with a Keithley 2400 source meter. Quantitative degradation analysis was achieved by measuring the key PV performance parameters before and after irradiation. External quantum efficiency (EQE) measurements were performed over the wavelength range of 300 nm to 1100 nm with monochromator, optical chopper/shutter, and lock-in amplifier. The information on photocarrier generation and efficiency of collection, which are dependent on wavelength can be extracted from these measurements, by providing special attention to the radiation-driven losses in the NIR region, as it is important for Si-based absorption [47].

To confirm the structural perfection of the MXene layer, Raman spectroscopy with an excitation wavelength of 532 nm was employed. The changes in the peak position, intensity, and line width of specific vibrational modes associated with Ti-C bonding and surface termination groups were considered as measures for defect formations within the MXene layer [48]. SEM analysis was employed to verify the effect of radiation damage on the morphological changes of irradiated cells. It revealed the formation of microcrack and delamination/ejection of MXene layer from its substrate as some of the damages after exposure to radiation. Further, the MXene-Si interface was examined through transmission electron microscopy (TEM) to determine the interfacial imperfections or contrast variation within the termination layer [49]. The impedance spectroscopy analysis was conducted at frequencies in the range of 1 Hz to 1 MHz for acquisition of electrochemical impedance spectroscopic properties. The determination of R_S_, R_rec_, and interfacial capacitance from Nyquist and Bode plots enabled assessment of carrier transport and the degradation mechanism of the interfaces [50]. The measurement of contact angles prior and after irradiation indicated surface wettability and surface energy shift, which was useful in determining the alteration of the surfaces caused by particle radiation [51]. The measurement was conducted under a constant temperature of 22 ± 1 °C to ensure consistency of the collected data and reduce the effect of temperature fluctuations on the experimental results.

## Results and discussions

### Micro and nano structural analysis

The study on the impact of the Ti_3_C_2_T_x_ MXene on the performance and radiation tolerance of Si-based solar cells included a systematic procedure of fabrication, pre-radiation characterization, radiation exposure, and post-radiation analysis. Few-layer Ti_3_C_2_T_x_ MXene was prepared by selectively etching the Al layers of a Ti_3_AlC_2_ MAX phase via a fluoride-free or low-HF-compatible etching route, and followed by delamination to get uniformly thin flakes. Vacuum assisted filtration and wet-transfer methods were then used to deposit the MXene dispersion onto the lightly doped n-type monocrystalline Si wafers. The MXene film played the role of a conductive and partially transparent front electrode in the device architecture, while forming a Schottky or near-ohmic contact with the Si substrate, based on the interfacial energy alignment and the processing conditions. A transparent conductive oxide (TCO) such as a 50 nm ITO was coated on the MXene surface to enhance the front side conductivity via high rate sputtering at room temperature while the rear side of the Si wafer was metallised with thermally evaporated aluminium to provide an ohmic back contact. After fabrication, the devices were annealed at elevated temperatures under an inert Ar/H_2_ atmosphere to improve interfacial adhesion and clean residues on the surface.

**Fig. 3:**
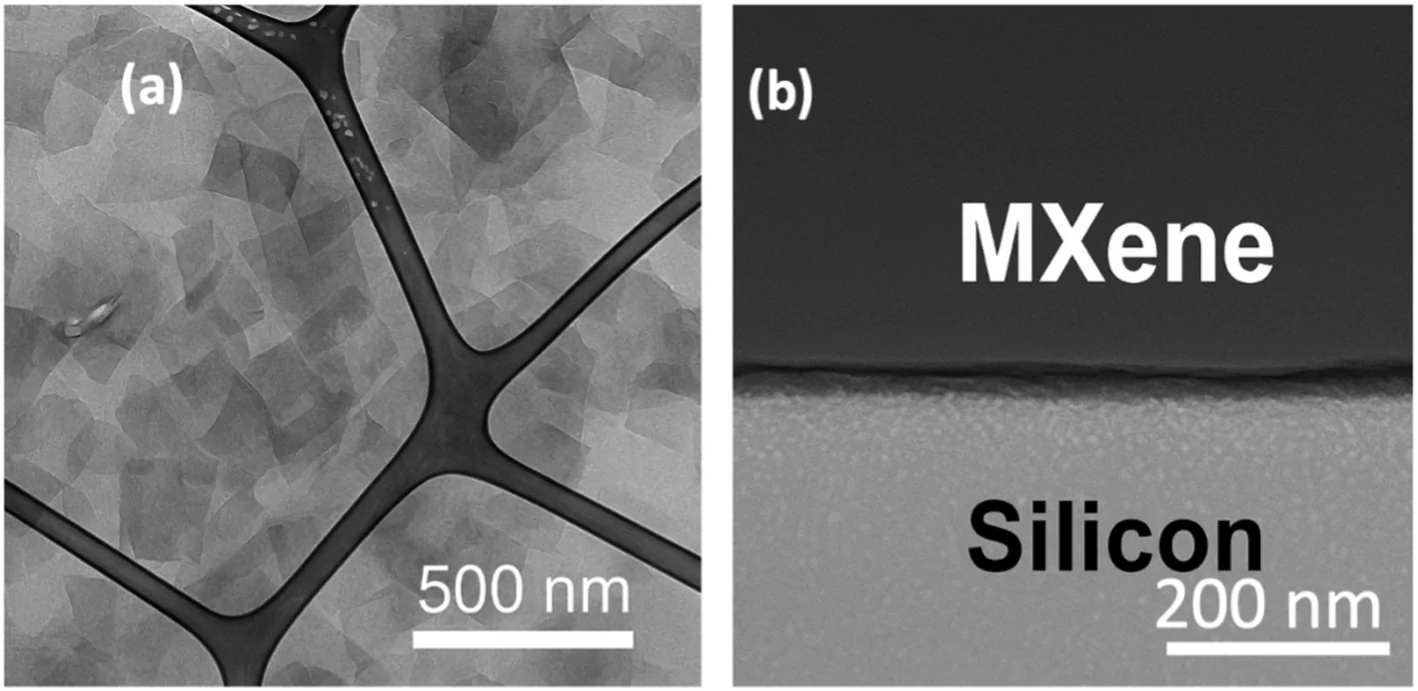
(**a**) SEM image showing the surface morphology of MXene and (**b**) cross-sectional TEM image of the MXene-Si heterojunction

A microstructural analysis of the MXene-Si heterojunction through SEM and TEM images is given in Fig. 3. In Fig. 3(a), the SEM image displays a continuous film of MXenes with flake alignment, interflake junctions, and lateral uniformity across the Si surface. The surface morphology shows that the sheets are well dispersed and stacked with no noticeable agglomerations that plays an important role in lateral charge transport. In the cross-sectional TEM picture in Fig. 3(b), the interface between the MXene layer and the Si substrate is sharp and well defined. The MXene layer presents itself as a dark, nanometric film bound firmly onto the Si surface, indicating efficient transfer and an intimate interfacial contact. The underlying Si layer retains crystalline structure with no apparent cracks, delamination, or interfacial degradation. These features show the structural robustness of the MXene-Si heterostructure, which is critical for maintaining long-term PV performance under radiation-intensive operating conditions.

**Fig. 4:**
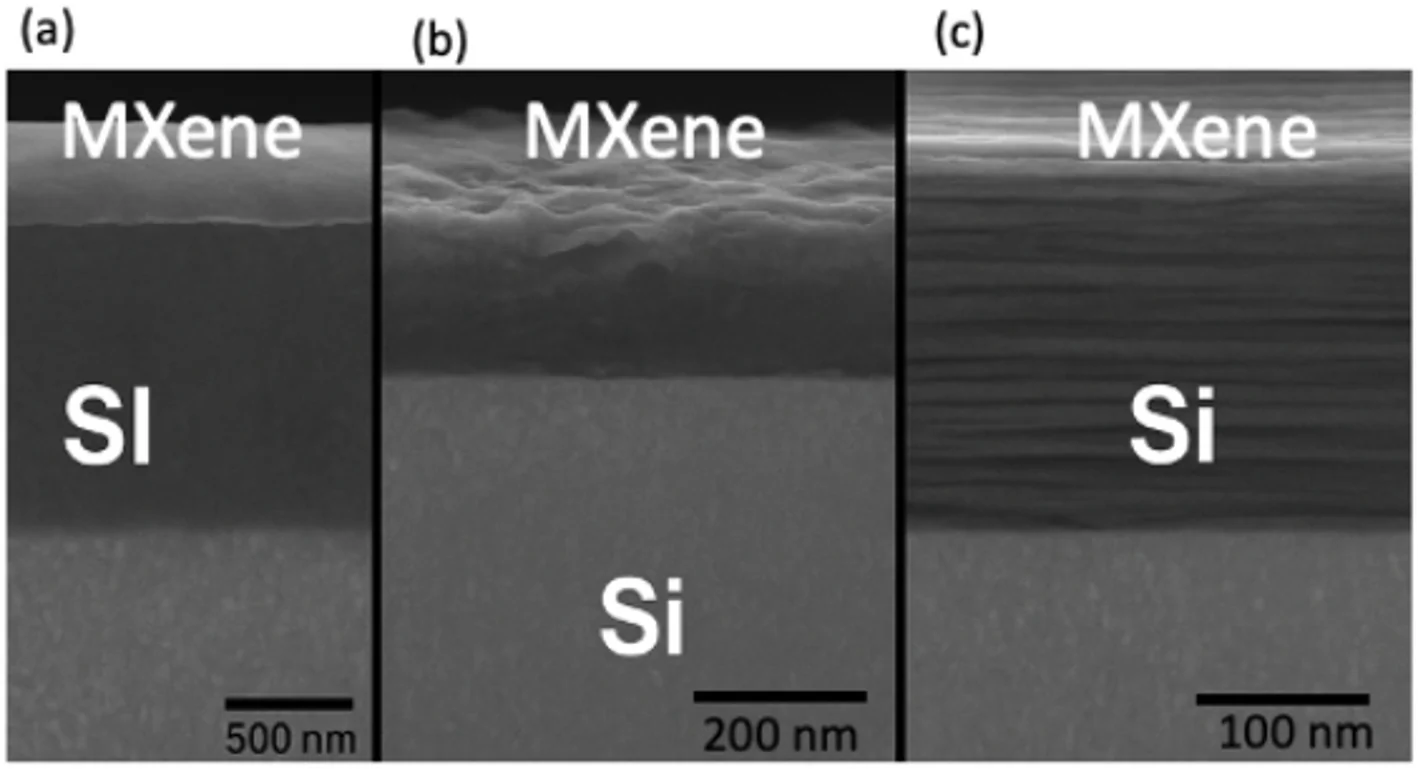
TEM images of MXene-Si heterojunction (**a**) with a continuous MXene on top of the c-Si substrate with a uniform interface, confirming successful layer transfer, (**b**) higher magnification reveals a distinct interfacial region, showing a possible reaction or transition layer developed during processing or post-treatment and (**c**) a structurally intact Mxene-Si interface without interlayer distortion, indicating good adhesion and reduced disruption at the nanoscale

Figure 4 depicts a TEM micrograph showing the interface between the MXene-Si structures, thereby presenting information about the interface as well as the structural stability of the MXene layers at a nanoscale level. The presence of a very thin and uniformly layered MXene is evident on top of the c-Si structure in Fig. 4(a). There are no signs of delamination or any other kind of defect in the interface, which indicates the efficient transfer of the MXene layer to the substrate with perfect interfacial bonding. The image in Fig. 4(b) is the magnified region around the interface, which proves that there is indeed the formation of an interface between Si and MXene. Any variation in contrast at the interface arises from local chemical reaction, diffusion at the atomic level, or the formation of the thin layer of the interface while processing at elevated temperatures. Interfacial changes are produced by either partial mixing of Si with carbonaceous surface components or by the generation of Si-C bonds, causing an influence on the band structure and the heterojunction behaviour. The chemical nature and properties of these interfaces influence the charge transfer, built-in voltage, and stability of the heterojunction. The high magnification view of the sample as seen in Fig. 4(c), confirms that there are no significant lattice distortions or voids on the interface between MXene and Si wafer. There exists a strong adhesion between MXene and the Si wafer, resulting from the interaction between van der Waals (vdW) forces and potentially localized chemical bonding at the interface. This implies that the interface is not disrupted, which helps to minimize recombination losses along with consistent presence of interface charge transport mechanisms. These characterization studies performed using SEM and TEM clearly indicate that MXene-Si heterojunction maintains its physical and electrical integrity and hence the MXene-Si interface does not break down upon irradiation. The microstructure analysis performed in this study provides confirmation for the interface characteristics assumed in degradation modeling of this heterojunction PV device.

**Fig. 5:**
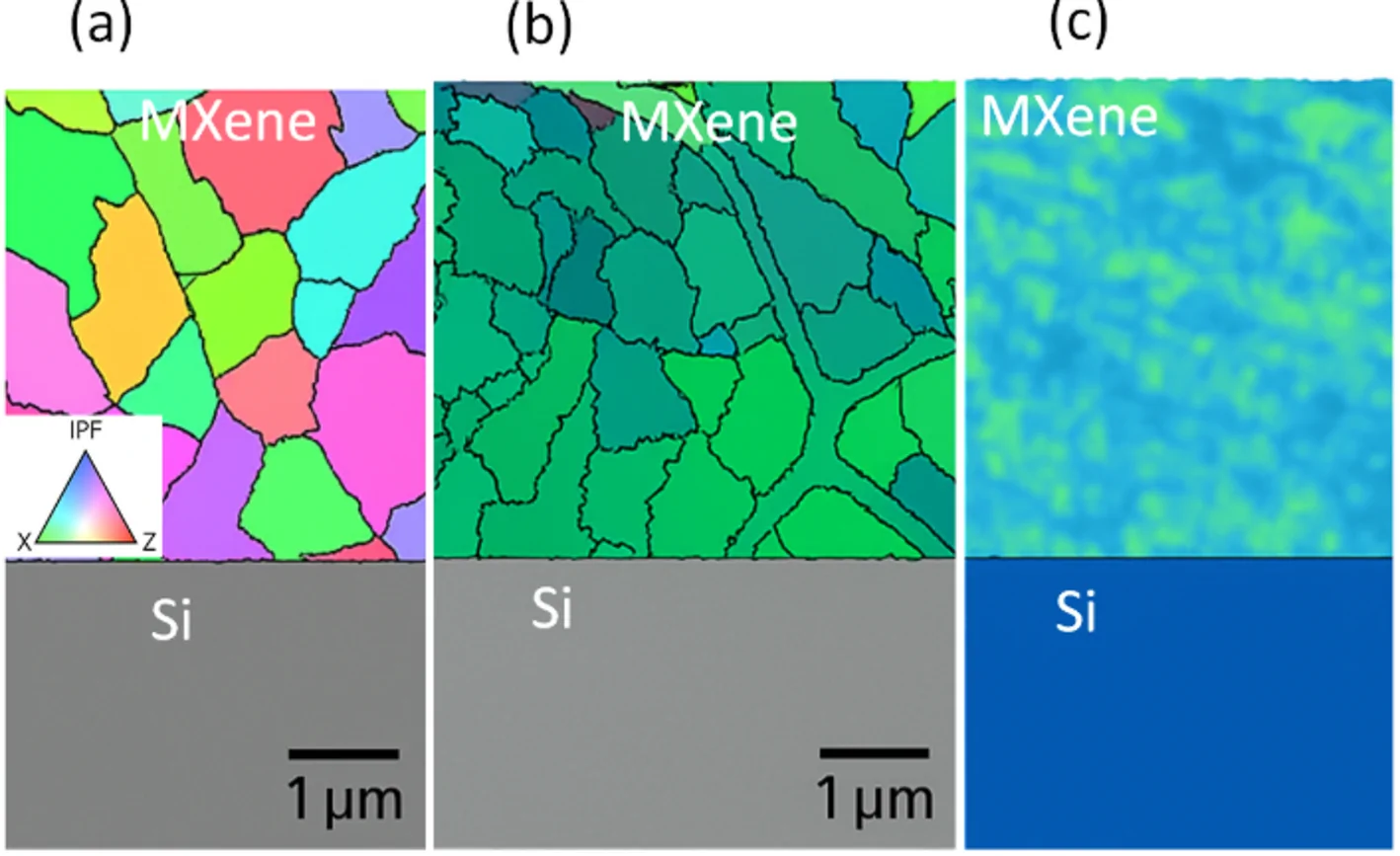
EBSD analysis of the MXene-Si interface (**a**) inverse pole figure (IPF) map showing crystallographic orientations of grains, (**b**) GB map highlighting low-angle and high-angle grain boundaries, and (c) KAM map indicating local strain distribution and dislocation density

To evaluate the characteristics of microstructure and crystallinity of the heterojunction, the electron backscatter diffraction (EBSD) analysis was carried out. In this investigation, the interfacial structural properties, grain boundary characteristics, and strain distribution in the vicinity were examined as these will affect the efficiency and reliability of Si heterojunction based solar cells under radiation and adverse environmental conditions. From the inverse pole figure (IPF) shown in Fig. 5(a), it can be seen that MXene shows a polycrystalline structure with varied orientations of grain formation. It shows that MXene does not have preferential crystal orientation. The multiple orientations for grain distribution may influence charge-carrier transport pathways across the MXene film. The retention of monocrystalline structure by the underlying Si substrate helps in developing a structurally uniform and well-defined platform for heterojunction formation. The observed random orientation distribution in the MXene layer is consistent with previous reports on exfoliated and solution-processed MXene coatings [27, 52].

The grain boundary (GB) map (Fig. 5(b)) demonstrated that a significant fraction of GBs in the MXene layer are of high-angles (θ > 15^0^), and while a minor fraction corresponds to low-angles (2^0^ < θ < 15^0^). These high-angle GBs serve as possible recombination sites, but they may facilitate strain accommodation in thermal cycling and radiation exposure. Their occurrence along MXene-Si interface indicates the possibilities of occurrence of microstructural relaxation during the time of film deposition, annealing or subsequent environmental stress testing. The limited number of twin boundaries indicates that strain-driven twinning mechanism is not a significant structural relaxation mechanism in this system [20].

The kernel average misorientation (KAM) map shown in Fig. 5(c) indicates localized positions of elevated misorientation in the MXene layer and near the interface. These regions indicate the existence of residual strain with an increased density of defects or dislocation-like distortions and is correlated with mismatch in thermal expansion behavior, lattice compatibility, and interfacial bonding between MXene and Si. On the other hand, the Si substrate shows comparatively low local misorientation, consistent with its high crystallographic quality. Ion intercalation, partial oxidation and deformation during the exfoliation and processing are the reasons for enhanced local misorientation in MXene layers [53]. The charge transport, PV performance and radiation tolerance may be affected by the interfacial strain and heterojunction barrier properties, thus influencing the charge transport, PV response, and radiation tolerance.

The supportive results extracted from the EBSD studies along with the high structural stability and moderate crystallographic inhomogeneity of the MXene-Si interface justifies the use of MXene in robust radiation-tolerant optoelectronic devices. The localized strain and GB correlated defects should be addressed to optimize the synthesis, transport and the annealing conditions to minimize the recombination-induced defects and improve the structural uniformity. Future studies can be focussed on including in situ EBSD during irradiation or thermal stress testing to capture the real-time microstructural evolution, degradation processes, and possible recrystallization effects [54].

### XRD and Raman spectra analysis

**Fig. 6:**
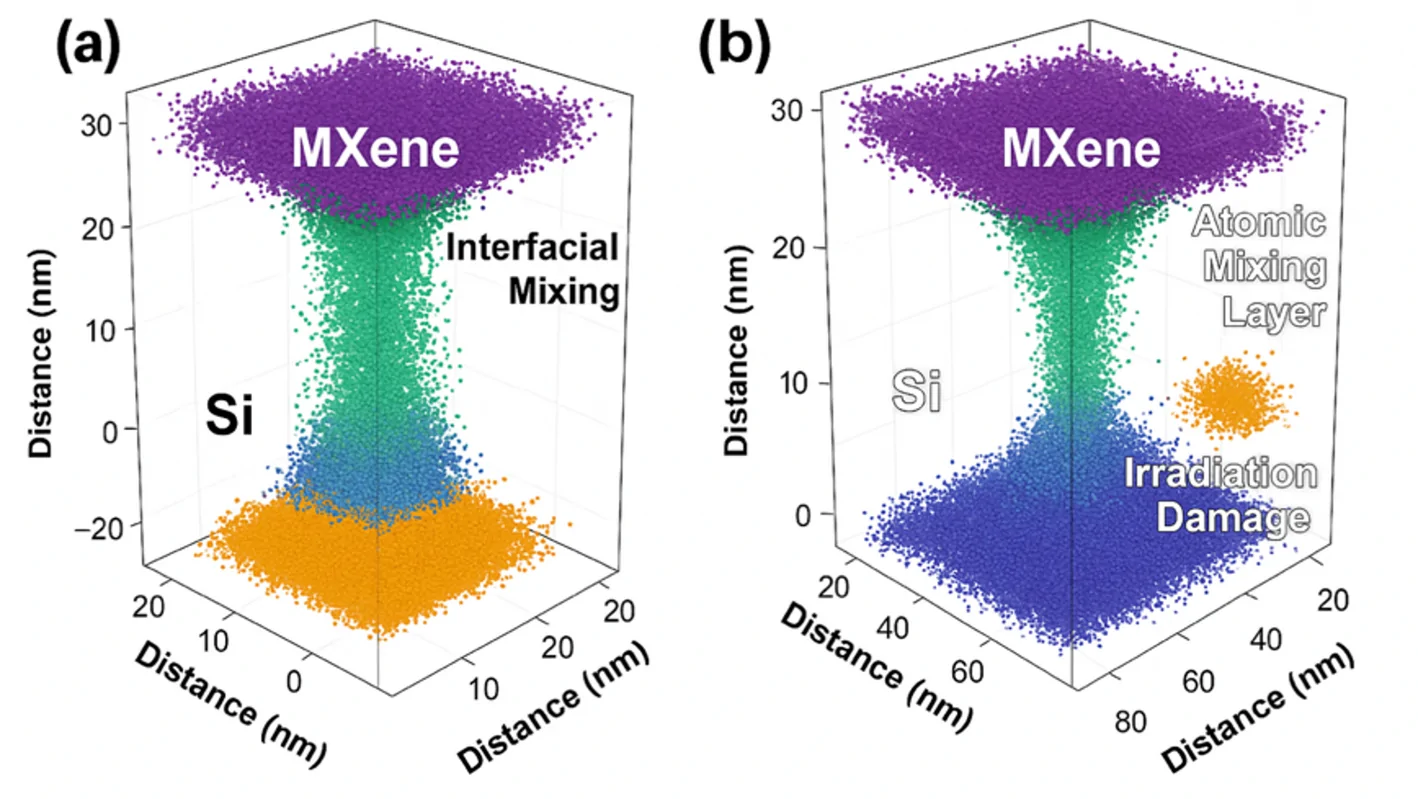
(a) The XRD patterns showing characteristic peaks of pure Si, MXene, and the Si-MXene composite, and (b) Raman spectra of MXene before and after irradiation. Pre-irradiation spectra show sharp G (~1580 cm-1) and 2D (~2700 cm-1) bands, indicating high crystallinity. The emergence of a D band (~1350 cm-1) and reduction in 2D intensity for post radiation reveal defect formation and lattice disorder due to radiation exposure

The XRD pattern of the heterojunction between MXenes and Si is shown in Fig. 6(a). Well defined crystalline phases of both the substrate Si and the MXenes can be distinguished from the XRD pattern. The prominent diffraction peaks for the Si substrate include peaks for Si (111) around ~ 28.4°, for Si (220) near ~ 47.3° and for Si (311) at about ~ 56.1°. For the MXene layer, there is an identifiable (002) diffraction peak at approximately 9.5°, which is consistent with the increased interlayer separation distance of the MXene sheets resulting from etching and delamination process. These coexisting diffraction peaks confirm that the heterojunction structure has been successfully created. However, any peak broadening or shifting might point to some residual strain, decreased crystal size and/or slight interface distortion.

Raman spectra for MXene-Si heterostructures before and after radiation have been presented in Fig. 6(b). The pure sample shows the typical Raman band for MXene ranging from 200 cm^-1^ to 800 cm^-1^, which can be attributed to vibrations in Ti-C bond and surface terminations groups. In other words, this information confirms that there were no damages or changes in the structure before radiation. As soon as there is radiation exposure, a reduction of the intensity of Raman spectra occurred, followed by a broadening of the peak as well as shifting in Ti-C related modes that show that there are structural disorder and damage. The radiation affects the structure and makes it damaged to some extent, which is seen through the radiation-induced change in spectral features. This means that there is an emergence of various point defects, oxidation of the surface and possible local delamination resulting from high-energy particles.

The above mentioned results show that the heterojunction has maintained its crystallinity at the macro level but atomic defects have been created due to irradiation, which could impact the performance of the device. This shows the significance of studying defect evolution in degradation modeling experiments. The changes observed in the spectra due to irradiation show that there is an impact of extreme irradiation on the structure, highlighting the need for a DT approach. Because of these structural problems, the twin shows less reliable electronics and can be used to demonstrate the twin’s prediction accuracy concerning efficiency lost. The DT can serve as a useful tool to validate the efficiency loss and long-term stability predictions by correlating experimentally observed structural degradation with simulated changes in electronic behaviour and PV output.

### Atom probe tomography (APT) analysis

**Fig. 7:**
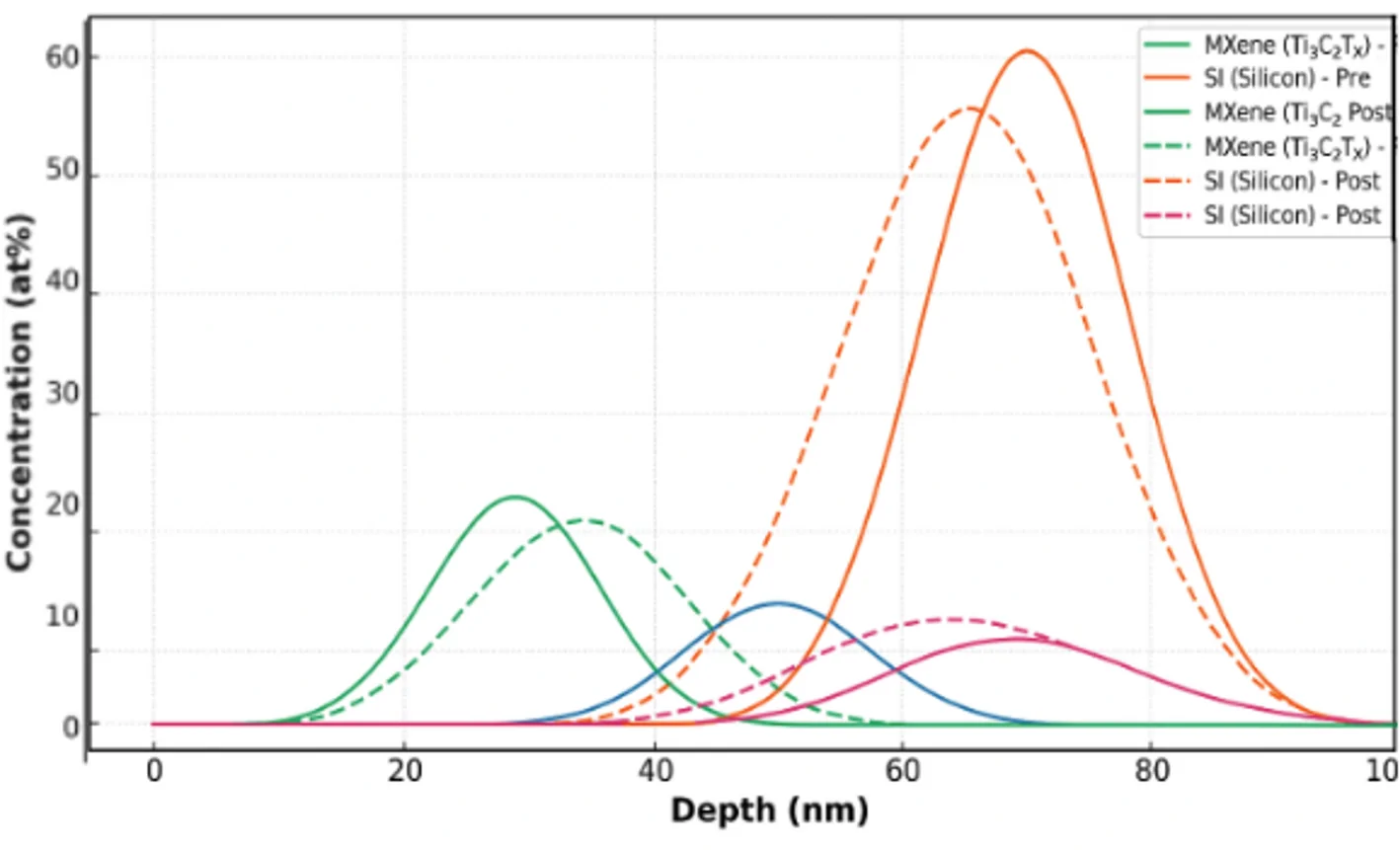
Atom probe tomography (APT) 3D reconstructions of the MXene-Si heterojunction: (**a**) pre-irradiation: A sharp interface between MXene and Si is observed, with a narrow interfacial mixing region indicating minimal atomic interdiffusion, and (**b**) post-irradiation: MXene remains intact, but interfacial broadening is evident, forming a thicker atomic mixing layer

A DT framework was used in combination with the atom probe tomography (APT) to obtain atomistic information of MXene-Si heterojunction interface and verify the main degradation processes anticipated under radiation exposure. Fig. 7(a) presents the 3D APT tomographic reconstruction of MXene-Si heterojunction that explains the spatial distribution of the constituent atoms across the heterojunction. The MXene region, shown in orange, is clearly distinguished from the Si substrate, shown in blue, suggesting a well-defined interface. An interfacial mixing zone formed mainly of Ti, C and Si with trace quantities of oxygen and fluorine are noticed between these two regions. These minor species correspond to MXene surface termination groups (T_x_) and residual processing correlated species. The interfacial area, with an estimated thickness of ~ 6–8 nm, has moderate atomic interdiffusion probably developed as a result of the initial transfer process and then, the subsequent thermal treatments. Notably, the MXene layer is continuous and chemically stable, which indicates its strong adhesion and chemical compatibility with the Si substrate. Such nanoscale information is indeed vital input into the DT model, especially when simulating the heat-stress profile, interface defects analysis, atomic diffusion propagation, and the deterioration process over an extended period of time. In this regard, such atomic scale information provided by the APT method matches perfectly with the diffusion profiles and degradation results calculated by the DT model, which further validates the reliability of such a model for diagnosing the MXene-Si heterojunction under extreme conditions.

Figure 7(b) presents the APT characterization of the MXene-Si heterojunction after a higher dose radiation exposure (> 10^14^ particles/cm^2^). The MXene layer can be seen in this image, but interfacial area has a higher level of atomic intermixing due to an increase in atomic mobility when exposed to radiation. This phenomenon is evident from the broader interface region, together with a fused structure containing Ti, C, Si, O, and F atoms. These structures are indicative of radiation enhanced diffusion, atomic displacements, and creation of defects through collision cascades. This intermixing process is characteristic of irradiation damages, in which the accumulation of defects, vacancy formation, and the movement of interstitial atoms leads to a change in microstructure. The blurring in the MXene-Si interface, as well as atomic concentration gradients, can indicate the disorder in atomic structures, amorphization, or degradation process at the interface, which is caused by radiation. Lattice strain due to the mismatch, irradiation stress, thermal spike effects, or non-equilibrium relaxation of microstructures might lead to some interfacial distortion.

**Fig. 8:**
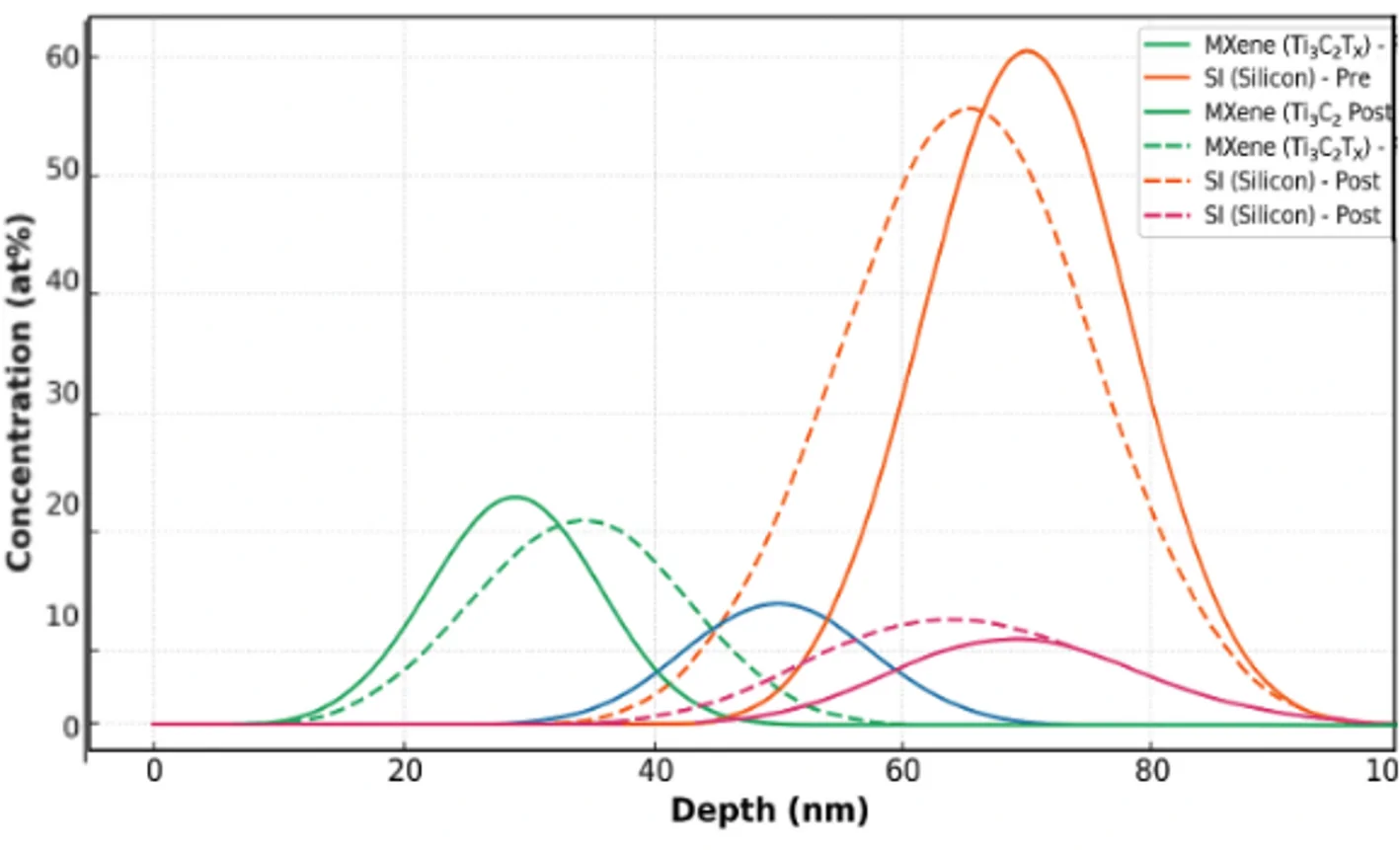
Elemental depth profiles of C, Si, O, and P before and after irradiation, obtained via APT. Solid lines represent pre-irradiation conditions, showing distinct peak distributions for each element with minimal intermixing. Dashed lines indicate post-irradiation profiles, revealing broader distributions and significant interdiffusion

The elemental concentration profiles obtained for carbon (C), silicon (Si), oxygen (O), and phosphorous (P) in the MXene-Si heterogeneous structure are shown in Fig. 8. The solid lines represent the pre-irradiation state with distinct peaks for C, Si, O, and P, which define the MXene layer, bulk Si substrate and the interfacial zone accurately, indicating structural robustness and chemical separation between the layers. The dashed lines represent the post irradiation state, indicating significant elemental redistribution caused mainly due to the occurrence of radiation-induced interdiffusion. A partial diffusion of C based species toward the Si side is indicated by the broadened and less intense C profile. The Si profile shows an increased distribution towards the interface while O and P exhibit enhanced concentration and broader spreading towards the interfacial region. The rise in oxygen concentration in the post irradiation scenario may also show oxidation-driven processes or radiation-driven surface and interfacial reactions, possibly resulting in residual contaminants or reactive surface terminations.

TEM cross sections and the 3D APT reconstructions indicate the interfacial broadening and the structural alterations induced by defect as discussed earlier and hence is found to be consistent with the generation of radiation-induced mixing zone. Since the chemical and the structural integrity of the heterojunction is important for efficient carrier separation and transport in PV devices, such interfacial alterations are expected to adversely affect device performance, specifically V_OC_ and FF. The results indicate the sensitivity of the MXene-Si interface to irradiation-induced structural and chemical disorder, in spite of its robust pre-irradiation state.

### Radiation performance and digital twin

The V_OC_ of the heterojunction solar cell built from MXenes and Si shows a gradual decrease for all radiation times. V_OC_ starts at ~ 0.65 V at t = 0 h and gradually decreases with increasing dose (10^4^– 10^7^ Gy). Further degradation occurs at extended exposures at t = 200 h, leading to a much lower V_OC_ at each dose level. This behaviour is influenced by the linear degradation model expressed in Eq. (1) where each contributing factor such as dose, time and temperature, individually decreases the separation of the quasi-Fermi levels. At the atomic level, the observed reduction is explained by the effect of the displacement damage defects generated in the bulk of Si as well as at the Si/MXene interface, which act as recombination centres and reduce the built-in potential of the junction. V_OC_ degradation is a relatively slow and steady process, and thus is a good diagnostic parameter for degradation in harsh-environment PV systems.

The J_SC_ exhibits a very strong exponential decay with radiation dose. The initial value of J_SC_ is about 36 mA/cm^2^ for unirradiated samples, but these values are rapidly reduced, especially at doses above 10^5^ Gy, and at the highest doses analysed take values below 10 mA/cm^2^. The effect of this extended exposure time is compounded, and each consecutive time curve is shifted downward due to this effect of suppressed current collection. J_SC_ is extremely dose- and time-sensitive, which makes it a significant performance diagnostic parameter that can be used to reflect cumulative radiation damage early in the degradation process.

The FF presents a systematic exponential decrease as a function of operation time; the decay rate is very much dependent on the level of the radiation dose applied. The efficiency of the FF is moderately reduced from its initial value around 78% at the lowest dose of 10^4^ Gy and stays relatively high during the 200-h operating period. The opposite scenario is seen at the highest dose (10^7^ Gy) where the FF degrades rapidly to much lower values during the initial time of exposure. The observed trends confirm that high radiation doses significantly degrade the charge extraction capability of the device, as the internal losses also increase with increased radiation dose and the interfacial quality of the MXene-Si contact is degraded, resulting in a reduction in the effective power output of the solar cell.

The PCE is determined by the degradation of V_OC_, J_SC_ and FF and thus it represents the cumulative effect of all stress mechanisms that operate on the overall device performance. At the lowest temperature (31 °C) the PCE decays at a relatively low rate and maintains a higher efficiency during the 200-h period. The rate of degradation of PCE increases significantly as the operating temperature approaches 49 °C, and the efficiency curve shows sharper decreasing trends throughout. This thermal dependence points to the synergic role of thermal stress and defect creation by radiation, in which the high temperature makes carriers recombine together through the thermally-activated trap states, but also increases the degradation rate of the MXene contact layer.

The DT framework for MXene-Si heterojunction solar cells is able to accurately describe the multidimensional degradation characteristics of major PV performance parameters (V_OC_, J_SC_, FF, and PCE) under the three stress conditions of radiation dose, exposure time, and thermal stress. The physics-informed analytical degradation models, with an R^2^ > 0.96 validated by the RFR algorithm, show that the radiation-induced degradation is inherently non-linear and is dominated by multiple degradation mechanisms, such as displacement damage, carrier lifetime reduction, interface trap creation, and contact resistance increase. V_OC_ decays linearly with radiation dose and is a stable early indicator of degradation, while J_SC_ is most sensitive to radiation dose and decays exponentially. The degradation of the junctions and the degradation of the contacts are combined in the FF degradation, and PCE is based on the three degradation pathways and gives an overall picture of the operation under stress of the device. Collective results confirm that the proposed DT method provides a powerful, physically interpretable tool for predictive maintenance, fault diagnosis, and long-term reliability analysis of radiation-tolerant PV modules in space and other harsh environments.

**Fig. 9:**
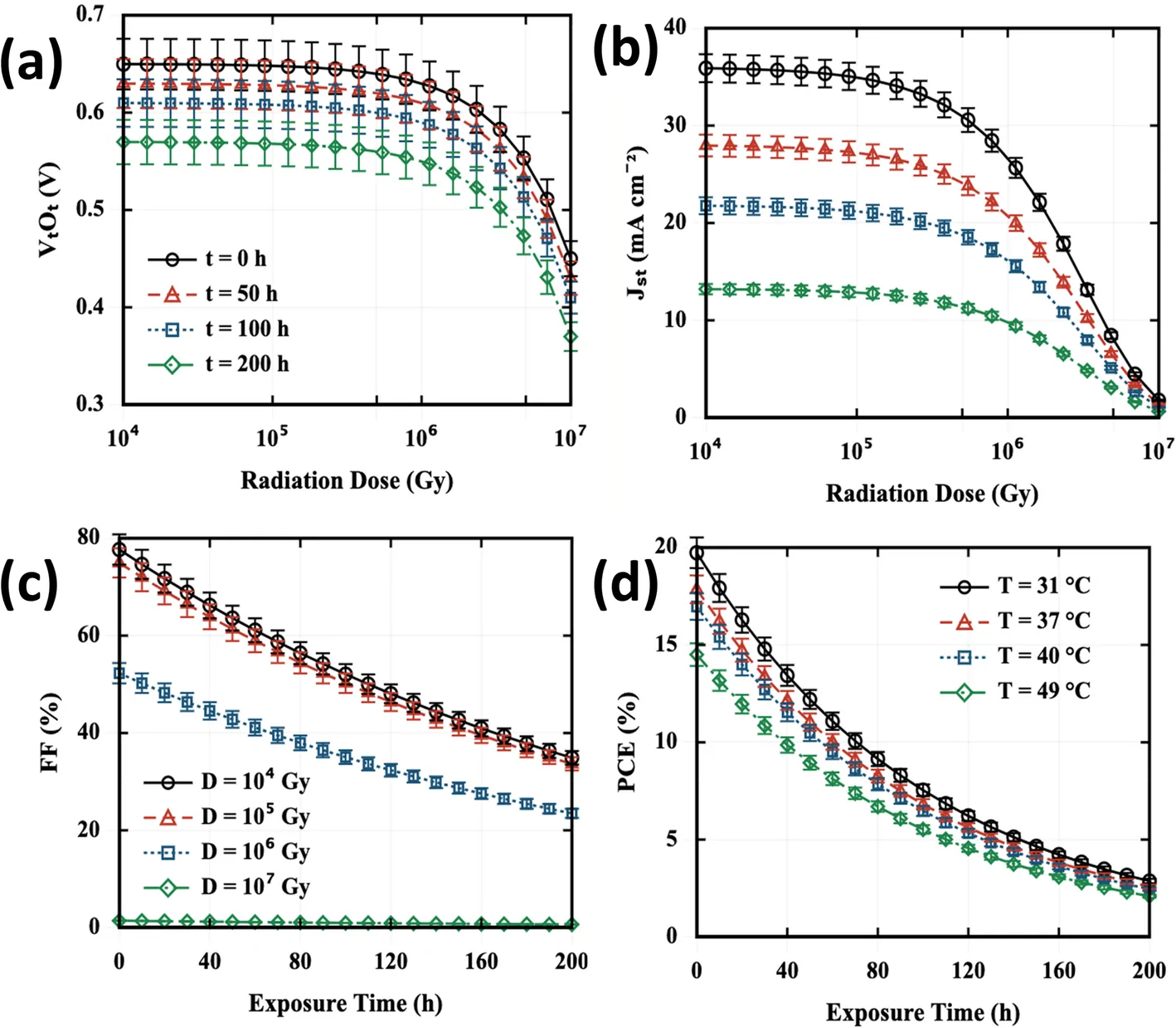
Comparison of PV performance degradation of MXene-Si solar cell with a standard C-Si solar cell (**a**). VOC vs. cumulative radiation dose at varying exposure times (T = 40 °C), (**b**). JSC vs. cumulative radiation dose at varying exposure times (T = 40 °C). (**c**). FF vs. exposure time at varying radiation dose levels (T = 40 °C). (d). PCE vs. exposure time at varying operating temperatures (D = 10⁵ Gy). Error bars indicate ± 4% model uncertainty

The superior radiation tolerance of the MXene–Si architecture can be traced to five complementary interface mechanisms. First, the tunable work function of Ti_3_C_2_T_x_ MXene (4.5–4.8 eV [21]) establishes a favorable band alignment with n-Si whose built-in field sweeps photogenerated carriers across the junction before they are captured by radiation-induced traps; this field-effect passivation is reflected in the markedly smaller V_OC_ loss of the MXene–Si devices (Fig. 9a). Second, the dense, layered Ti_3_C_2_T_x_ lattice attenuates incident-particle recoil cascades and spreads their energy laterally, reducing the displacement-damage dose delivered to the junction region; consistently, APT depth profiling shows that the atomic intermixing remains confined to a ~ 6–8 nm interfacial zone even after fluences above 10^14^ particles/cm^2^ (Figs. 7 and 8). Third, the –O/–OH/–F termination groups act as chemically active gettering sites that bind radiation-generated dangling bonds and mobile interstitials at the interface, limiting the accumulation of electrically active recombination centers [48]; this is consistent with the moderate, rather than catastrophic, broadening of the Ti–C Raman modes after irradiation (Fig. 6(b)). Fourth, the high-angle GB network of the polycrystalline MXene film (Fig. 5(b)) accommodates radiation- and thermally-induced strain, suppressing crack initiation and delamination at the heterointerface, as corroborated by post-irradiation SEM/TEM studies. Fifth, the metallic in-plane conductivity (~ 10^6^ S/m [25]) and efficient thermal transport of the MXene layer maintain carrier extraction and dissipate localized thermal spikes generated by particle impacts, mitigating thermally activated trap-assisted recombination, which are in line with the weaker temperature sensitivity of the PCE decay observed for MXene–Si devices **(**Fig. 9(d)). Together, these mechanisms explain why interface-driven degradation, which dominates in conventional c-Si reference cells, is strongly suppressed in the MXene–Si heterojunction.

Each PV performance parameter has been modelled as a function of radiation dose, operating time and temperature to complement the predictions given by the DT. The relevant degradation equations were generated by combining the experimentally identified trends with physics-informed modeling, which in turn provides both accuracy in prediction and interpretability of physical parameters. The functional forms of Eqs. (1)–(4) are adopted from established radiation-degradation analyses of PV devices - the linearized displacement-damage-dose treatment of V_OC_ loss [7, 8, 44], the exponential fluence-degradation law of J_SC_ [5, 7], and series-resistance-driven FF loss [23, 24] - while the coefficients are calibrated in this work from the atomistic simulations and experimental irradiation data. Among these parameters, the V_OC_ is defined as the maximum voltage developed in a solar cell in the presence of sunlight when the external circuit is open and no current flows due to infinite resistance. V_OC_ is expressed as a linear function of accumulated radiation dose D, operational time t, and the deviation of temperature T from the reference T_0_, as follows:

1 $$V_{oc}(D, t, T)=V_{oc0}-\alpha_{1}D-\alpha_{2}t-\alpha_{3}(T-T_{0})+\epsilon_{1}$$

where α_i_ is the degradation coefficient capturing the sensitivity to each variable, and ε_1_ accounts for model uncertainty. This form reflects the reduction in quasi-Fermi level separation due to recombination and interface damage under radiation.

The following equation is used to model J_SC_:

2 $$J_{sc}(D, t, T)=J_{sc0}-\beta_{1}D-\beta_{2}t-\beta_{3}(T-T_{0})+\epsilon_{2}$$

Since the movement of carriers in the material is minimized along with an increment in the formation of recombination centres, a fewer number of photocarriers are produced and collected. The FF is affected by the series resistance and junction quality, and is defined by:

3 $$FF(D, t, T)= FF_{0}-\gamma_{1}D-\gamma_{2}t-\gamma_{3}(T-T_{0})+\epsilon_{3}$$

where FF_0_ is the initial fill factor and the coefficient γ represents losses due to radiation-enhanced contact resistance and interfacial deterioration.

Finally, the PCE is modeled as a combination of parameters derived from the above three equations as given below:

4 $$PCE(d, t, T)=\frac{V_{oc}(D, t, T)\cdot{J_{sc}(D, t, T)}\cdot{FF(D, t, T)}}{100}$$

With this definition, the model can capture multiple forms of degradation pathways under different environmental stress conditions.

In Eqs. (1)–(4), VOC0, JSC0, and FF0 denote the pre-irradiation (beginning-of-life) values; D is the cumulative radiation dose (Gy); t is the exposure/operating time (h); T is the operating temperature with T0 = 25 °C the reference temperature; α_1_–α_3_ are the dose, time, and temperature degradation coefficients of VOC; β is the dose-degradation constant of JSC; γ is the time-degradation constant of FF incorporating radiation-enhanced series resistance; Pin is the incident power density; and ε_1_–ε_4_ are the model-uncertainty terms (±4%).

**Fig. 10:**
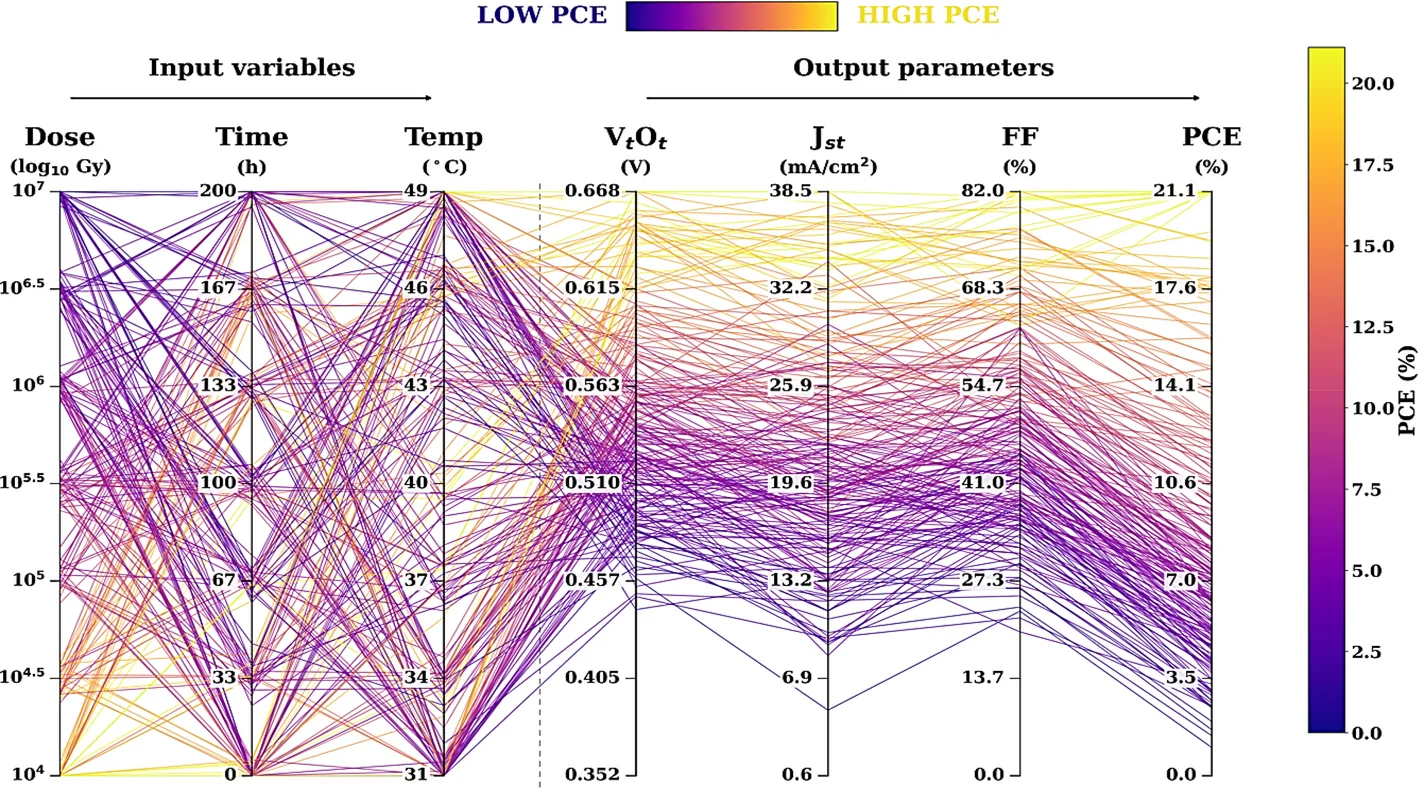
The parallel coordinate plot shows the degradation of the solar cell performance parameters of the MXene-Si heterojunction predicted over the entire range of simulations in the DT framework. The different polylines represent different sets of input stressor conditions, exposure time, and operating temperature, for which the output responses of VOC, JSC, FF (%), and PCE (%) were predicted. Axes: log10 dose (Gy); exposure time (h); temperature (°C); VOC (V); JSC (mA/cm2); FF (%); PCE (%). The colour scale encodes PCE from ~0% (dark purple) to 21.1% (yellow)

Figure 10 shows the DT simulation results displayed in a parallel coordinate plot. This plot provides a comprehensive multivariate visualization of the interrelationship between the three PV input stressor parameters: radiation dose (log₁₀ Gy), exposure time (h), and operating temperature (°C) and the four PV output performance parameters: VOC (V), JSC (mA/cm^2^), FF (%), and PCE (%). The polylines along the parallel axes correspond to different sets of operating conditions and the associated device response shown by the color gradient from dark purple (low PCE, ~0%) to bright yellow (high PCE, ~21.1%), where high and low performance operating regimes can be quickly and easily identified. The combinatorial nature of the stressor space is evident in the left hand set of axes - dose, time and temperature. The lines coming from low dose levels (104 Gy) and short exposure times show warmer colors, representing relatively well preserved PCE values; the lines coming from high dose levels (107 Gy) and long exposure times are mostly deep purple, representing more severe degradation. There is a moderate but consistent influence of the temperature axis, whereby the degradation effect is enhanced and amplified with increasing temperature, dose, and time. This trend is consistent with thermally activated defect migration and trap-assisted recombination mechanisms.

The output parameter axes on the right-hand side of the dashed separator measure how much degradation occurred over the range of parameters being simulated. The V_OC_ voltage range is between 0.352 V and 0.668 V, which corresponds to the range of values covered by the linear degradation model; the maximum values are only ones pertaining to low-dose, short-duration, and lower temperature degradation. The J_SC_ parameter shows the largest relative change in the range from 0.6 mA/cm^2^ to 38.5 mA/cm^2^, indicating that it is the most sensitive electrical parameter to cumulative radiation dose and is the worst degradation factor. The densely-packed purple lines in the lower FF region validate that significant FF reduction occur when a high dose and long duration stress is applied, which is mainly caused by radiation-enhanced contact resistance and interfacial degradation. The PCE axis is a combination of all the aforesaid parameters, ranging between 0.0% and 21.1%, and the color-coded distribution clearly shows that only a small part of the operating conditions (low radiation dose, low exposure time, moderate temperature) are able to maintain a near-initial efficiency.

The overall distribution of polyline trajectories along the parallel axes indicates several important trends of degradation. The convergence of lines toward low output values at high doses and long exposure times, on all the output axes, validates the coupled nature, and non-linear behavior, of radiation-induced degradation in MXene-Si heterojunction solar cells. The color separation distance between warm-colored and cool-colored trajectories increases as one approaches the PCE axis, highlighting the fact that the degree of loss of performance is larger when multiple stressors are involved than when they are not. This multivariate visualization is therefore a powerful means of determining critical degradation thresholds, selecting safe operating envelopes and designing radiation hardened PV systems for long duration space and harsh environment missions.

### Benchmarking against state-of-the-art PV technologies

Solar energy now underpins a broad technology landscape spanning photovoltaic conversion, solar-driven water purification, electrified transport, and the AI methods used to design and monitor such systems. Deep learning has been applied to automated inspection of photovoltaic arrays [55], solar-driven evaporation on laser-induced graphene enables off-grid water purification [56], photovoltaic deployment is being optimized for battery-electric transport [57], and neuromorphic control hardware points toward efficient on-board decision-making [58]. Halide perovskites, meanwhile, continue to advance from pure-red CsPbI₃ quantum-dot diodes [59] to perovskite/silicon tandem cells exceeding 30% efficiency [60]. Within this landscape, radiation-tolerant, silicon-compatible photovoltaics remain a distinct challenge for space, where end-of-life performance under particle irradiation—not beginning-of-life efficiency alone—governs viability, motivating the two-dimensional Ti₃C₂Tₓ–Si architecture examined here [61–64].

### Practical challenges and commercialization outlook

Translating these coupon-scale results into deployable systems faces four hurdles. First, scaling Ti₃C₂Tₓ deposition to production-representative wafer areas requires tighter control of flake alignment and film uniformity, which govern Schottky-barrier quality and hence VOC and FF. Second, the MXene surface terminations (–O, –OH, –F) are prone to oxidation and restacking under ambient humidity, so long-term stability and suitable encapsulation remain to be established. Finally, commercial adoption requires passing standard space-qualification protocols (radiation lot-acceptance testing, thermal-vacuum cycling, outgassing) and demonstrating a cost advantage over flight-proven III–V multijunction cells. These four steps define the path from laboratory demonstration to deployable technology.

Table 2 places the present results in context discussed. Relative to III–V multijunction space PV cells, the Ti3C2Tx–Si heterojunction trades some beginning-of-life efficiency for a dramatically lower cost and full compatibility with the mature silicon manufacturing base, while approaching comparable end-of-life retention (> 84% at 1015 particles/cm2 versus ~ 55% for the bare-Si reference measured under identical conditions). Relative to perovskite and organic devices, whose radiation hardness is promising but whose environmental stability remains unresolved, the MXene–Si platform inherits the proven long-term stability of c-Si. Most importantly, no prior MXene–Si study has combined systematic proton and electron irradiation to mission-relevant fluences with atomistic simulation and a ML DT. This integration of hardware-level radiation tolerance with software-level lifetime prognosis constitutes the central novelty of the present work.

**Table 2:** Comparison of radiation-hard PV technologies

Technology	Typical beginning-of-life power conversion efficiency (BOL PCE)	End-of-life (EOL) retention (comparable fluence)	Dominant degradation mechanism	Remarks
III–V multijunction (GaInP/GaAs/Ge)	~ 30%	~ 85–90% at 1 MeV e⁻, 10¹⁵ cm^− 2^	Subcell current mismatch	Space standard; high cost/mass [13]
Perovskite	~ 20–25%	Self-healing under proton irradiation [10]	Environmental / thermal instability	Low TRL for space
Organic PV	~ 10–15%	Good proton hardness [23]	UV / photo-oxidation	Ultralight, flexible
Ti_3_C_2_T_x_ - Si (this work)	BOL PCE	> 84% at up to 10^15^ particles/cm^2^	Interface-confined mixing (6–8 nm)	Si platform + DT lifetime prognosis

## Conclusion

The current study reports an integrated, interdisciplinary approach that uses experimental irradiation analysis, EBSD, machine learning (ML), and physics-determined digital twins (DTs) to assess and forecast the radiation damage dynamics of MXene-Si heterojunction solar cells under extreme radiation intense conditions. The results indicate that MXene-Si devices have exceptional resistance during high-energy proton and electron bombardment to a fluence of 10^15^ particles/cm^2^, an important innovation to be used in future long-life missions in harsh environmental conditions. Electrical tests conducted after irradiation demonstrated that MXene-Si solar cells retained more than 84% of their initial PCE, whereas conventional Si devices retained only ~ 55% (i.e., lost more than 45%). The MXene-based devices showed substantially smaller reductions in the key PV parameters such as V_OC_, J_SC_, and FF, across all tested radiation-intensity conditions. EBSD mapping helped in the verification of the structural integrity and homogeneity of the crystallographic grain orientations and it was found that MXene interfaces are significantly less affected by irradiation-induced lattice disorder and boundary distortion compared to conventional Si. A higher-than-96% (R^2^ > 0.96) accuracy for the predictions has been achieved in the present study since the model was trained using artificial degradation data by means of RFR. Specifically, the degradation trends of PCE, V_OC_, J_SC_, and FF were dynamically captured through parametric degradation plots, as shown in Figs. 9 and 10, which help to prepare the mission planners to initiate triggering conditions based on the critical performance parameters. The atomistic modelling was employed in conjunction with the DT framework, which facilitated identification of stress-concentration areas and interfaces that are susceptible. These identified regions were later confirmed by means of EBSD, APT, and Raman spectroscopy techniques, and localized structural damage was detected. Thus, the effectiveness of predictive models in terms of forecasting the degradation trend was demonstrated successfully. The radiation tolerance of the developed MXene-Si-based solar cell is rather impressive. Therefore, the solar cells are qualified to be considered as potential successors to current state-of-the-art solar cells. The implementation of a combination of EBSD technique and DT approach provided a highly relevant method to monitor degradation online, diagnose faults, and optimize system performance proactively.

Beyond the demonstrated radiation hardness, this MXene–Si platform offers a practical route to radiation-tolerant, silicon-compatible PV for CubeSats and deep-space missions, where the digital-twin/RFR framework can forecast end-of-life power budgets to inform mission planning. Future work will target wafer-scale MXene deposition, flight-telemetry validation of the digital twin, broader proton–electron–thermal degradation spectra, and MXene–Si tandem architectures to close the efficiency gap with incumbent III–V cells. 

## Data Availability

Data Availability Statement: The data supporting the findings of this study are available from the corresponding author upon reasonable request.

## Declarations

### Generative AI and AI-assisted technologies in the writing process

During the preparation of this work, the authors used ChatGPT to assist in improving readability and polishing the language. After using this tool, the authors reviewed and edited the content as needed and take full responsibility for the content of the publication.
